# Supplementary material for: Efficacy, Pharmacokinetics, and Toxicity Profiles of a Broad Anti-SARS-CoV-2 Neutralizing Antibody
Source: Viruses. 2023 Aug 14;15(8):1733. doi: 10.3390/v15081733 (PMC10459920; doi:10.3390/v15081733)
Supplement: Supplementary file 1 [file viruses-15-01733-s001.zip › viruses-2554688-supplementary.pdf]

Supplementary Material

Efficacy, pharmacokinetic and toxicity profiles of a broadly SARS-CoV2 neutralizing antibody

Silvia Godínez-Palma[1,2,†], Edith González-González[1,2,†], Frida Ramírez-Villedas[1,2], Circe Garzón-Guzmán[1,2], Luis Vallejo-Castillo[1,2], Gregorio Carballo-Uicab[1,2], Gabriel Marcelín-Jiménez[4], Dany Batista[4], Sonia M. Pérez-Tapia[1,2,3*] and Juan C. Almagro[1,2,5*]

Affiliations:

1 Unidad de Desarrollo e Investigación en Bioterapéuticos (UDIBI), Escuela Nacional de Ciencias Biológicas, Instituto Politécnico Nacional, Mexico City 11340, Mexico.

2 Laboratorio Nacional para Servicios Especializados de Investigación, Desarrollo e Innovación (I+D+i) para Farmoquímicos y Biotecnológicos, LANSEIDI-FarBiotec-CONACyT, Mexico City 11340, Mexico.

3 Departamento de Inmunología, Escuela Nacional de Ciencias Biológicas, Instituto Politécnico Nacional (ENCB-IPN), Mexico City 11340, Mexico.

4 Pharmometrica Analytical & Statistics Unit, Av. Eje 5 Norte 990, Edificio "C" planta baja, 02230 Mexico City, Mexico.

5 GlobalBio, Inc., 320 Concord Ave, Cambridge, MA 02138, USA.

[†] These authors contributed equally to this work.

* **Correspondence:**

Sonia M. Pérez-Tapia
Email: sperezt@ipn.mx
Phone number.: + (52) 55-5498-6692 or + (52) 55-5729-6000; Ext. 62543.
Juan C. Almagro
Email: juan.c.almagro@globalbioinc.com
Phone number.: + (617) 710-4487.

List of Figures and Tables:

Figure S1. Calibration curve of ELISA.

Table S1. ELISA performance validation parameters.

Tables S2. Serum IgG-A7 concentration values in CD-1 mice i.v. administered with the single dose of 100 mg/kg.

Tables S3. Serum IgG-A7 concentration values in CD-1 mice i.v. administered with the single dose of 200 mg/kg.

Table S4. Hematology summary of CD-1 mice 14 days after i.v. treatment with IgG-A7.

Table S5. Blood chemistry summary of CD-1 mice 14 days after i.v. treatment with IgG-A7.

Table S6. Mouse TCR.

Table S7. Human TCR.

ELISA Validation:

The parameters of the ELISA validation were established according to the suggested in the M10 ICH Harmonized Guideline for validation of bioanalytical methods and sample analysis. The parameters evaluated included selectivity, specificity, precision, accuracy and dilution linearity and Hook effect.

An IgG-A7 calibration curve was generated by fitting the raw data to a four-parameter (4PL) model. From this curve, the linear and quantification intervals were established, as well as the detection (DL) and quantification (DQ) limits. Specificity was determined as the ability to detect different anti-RBD antibodies, in addition to IgG-A7, including CB6, Bebtelovimab. The anti-lysozyme D1.3 antibody was used as negative control. Selectivity was established through the detection of IgG-A7 in different matrices (MPBST 3%, serum, hemolyzed serum and plasma). The precision was evaluated through the coefficient of variation (CV) of the results obtained intra- and inter-assay.

Accuracy was assessed by the ability to detect IgG-A7 at concentrations similar to those expected in serum samples from previously treated mice. Untreated serum samples were spiked with a 1 mg/mL spike of IgG-A7 (Average concentration estimated from the amount of antibody administered to the mice). Subsequently, serial dilutions was performed to evaluate the recovery by comparing the calculated concentration with the percentage of recovery. Further, the dilutional linearity was

determined to rule out a Hook effect and the linear range of the dose-response curve was selected to obtain the measurement interval IgG-A7 in the serum samples.

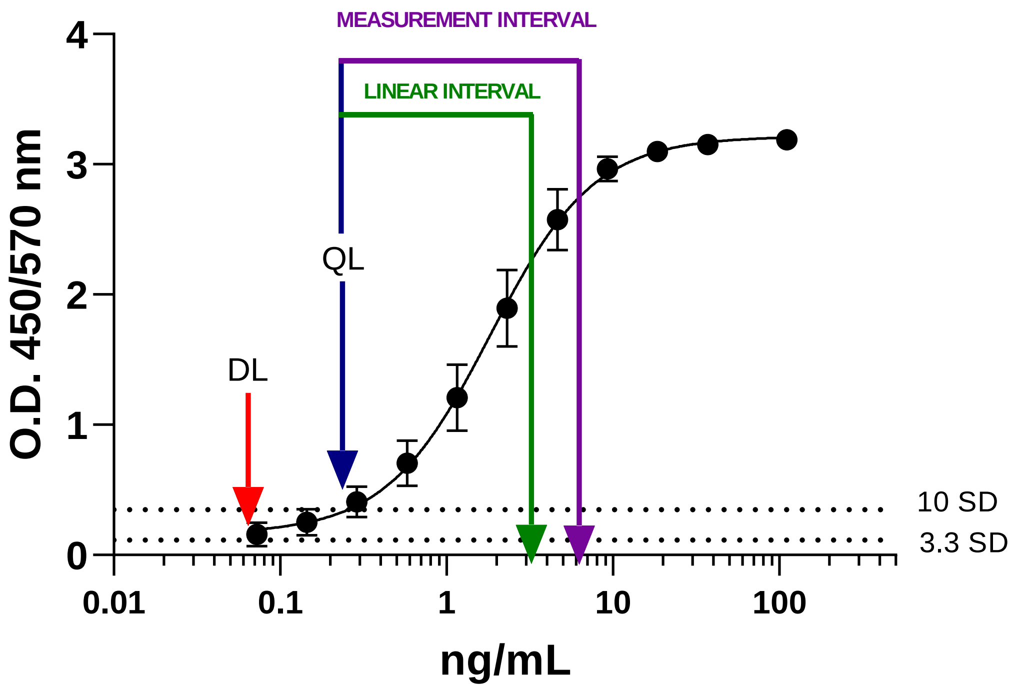

Figure S1. ELISA Calibration Curve. DL, Detection Limit; QL, Quantification Limit

Table S1. ELISA performance for the quantification of IgG-A7 in serum.

Attributes	Parameters	Value
**Calibration Curve**	Linearity	$R^2 \geq 0.9$
	Detection Limit (DL)	0.07 ng/mL
	Quantitation Limit (QL)	0.29 ng/mL
	Dynamic Range	0.07 – 100 ng/mL
**Precision**	Repeatability (%CV)	0.3 – 15.67 %
	Intermediate precision	0.84 – 11.42 %
**Specificity**	Anti-SARS-CoV-2 antibodies	Curve dose response $R^2 \geq 0.9$
	Non-related antibodies to SARS-CoV-2	Non-curve dose-response
**Selectivity**	Matriz signal (serum, hemolyzed serum, plasma)	Signal similar to blank
**Accuracy**	Recovery (%)	108. 9 – 125.5 %

PK raw data:

Tables S2. Serum IgG-A7 concentration values in CD-1 mice administered with the single dose of 100 mg/kg.

	TIME (h)								
	1	2	4	8	24	48	96	144	336
**M1**	664.54	609.21	391.85	495.91	*	290.88	316.22	322.57	173.02
**M2**	561.79	*	501.94	294.08	239.13	333.74	253.40	225.33	194.57
AVERAGE	613.17	609.21	446.90	395.00	239.13	312.31	284.81	273.95	183.80
SD	72.66	---	77.85	142.72	---	30.31	44.42	68.76	15.24
CV (%)	11.85	---	17.42	36.13	---	9.70	15.60	25.10	8.29
**F1**	651.17	562.09	460.63	435.66	355.99	347.2	316.7	268.62	178.72
**F2**	655.63	640.85	310.94	351.25	313.55	246.86	*	152.34	185.07
AVERAGE	653.40	601.47	385.79	393.46	334.77	297.03	316.70	210.48	181.90
SD	3.15	55.69	105.85	59.69	30.01	70.95	---	82.22	4.49
CV (%)	0.48	---	27.44	15.17	---	23.89	---	39.06	2.47
**M & F**									
AVERAGE	633.28	604.05	416.34	394.23	302.89	304.67	295.44	242.22	182.85
SD	47.98	39.63	83.66	89.32	59.15	45.41	36.41	71.92	9.24
CV (%)	7.58	6.56	20.09	22.66	19.53	14.90	12.32	29.69	5.05

M: Male. F: Female. Values are reported in μg/ml. * Outliers.

Tables S3. Serum IgG-A7 concentration values in CD-1 mice administered with the single dose of 200 mg/kg.

	TIME (h)								
	1	2	4	8	24	48	96	144	336
**M1**	1187.68	2153.97	1043.57	1737.84	600.22	834.15	664.92	825.36	142.36
**M2**	1408.19	*	1633.01	1483.40	561.38	1069.37	605.65	*	147.49
AVERAGE	1297.94	2153.97	1338.29	1610.62	580.80	951.76	635.29	825.36	144.93
SD	155.92	---	416.80	179.92	27.46	166.33	41.91	---	3.63
CV (%)	12.01	---	31.14	11.17	4.73	17.48	6.60	---	2.50
**F1**	1392.06	1117.54	1178.76	838.08	745.83	619.57	663.44	422.12	420.45
**F2**	1459.31	1218.78	1038.67	1148.52	1015.34	490.43	711.51	551.49	440.82
AVERAGE	1425.69	1168.16	1108.72	993.30	880.59	555.00	687.48	486.81	430.64
SD	47.55	71.59	99.06	219.51	190.57	91.32	33.99	91.48	14.40
CV (%)	3.34	6.13	8.93	22.10	21.64	16.45	4.94	18.79	3.34
**M & F**									
AVERAGE	1361.81	1496.76	1223.50	1301.96	730.69	753.38	661.38	599.66	287.78
SD	119.57	571.40	280.62	392.28	205.70	253.92	43.34	205.89	165.18
CV (%)	8.78	38.18	22.94	30.13	28.15	33.70	6.55	34.33	57.40

Hematological analysis:

Ten hematological parameters were measured, and the values were compared with reference ranges of CD-1 mice [https://www.criver.com/products-services/find-model/cd-1r-igs-mouse?region=3616.]. The results showed no significant differences between the treated groups with single doses of 100 and 200 mg/kg of IgG-A7 and control group (no antibody). The values are reported in the table below.

Table S4. Hematological parameters summary in CD-1 mice, 14 days after i.v. administration of IgG-A7.

Hematological parameter	Sex	Reference values #	Control	100 mg/Kg	200 mg/Kg
**HCT** (%)	M	49.97 ± 7.67	49.00 ± 2.0	46.00 ± 2.0	47.00 ± 2.0
	F	49.43 ± 6.70	48.00 ± 2.0	46.00 ± 2.0	48.00 ± 1.2
**HGB** (g/dL)	M	14.67 ± 2.21	14.68 ± 0.62	15.30 ± 0.56	14.70 ± 0.86
	F	14.55 ± 2.05	14.60 ± 0.87	15.20 ± 0.85	14.70 ± 0.62
**RBC** (M/μL)	M	8.93 ± 1.37	9.00 ± 0.62	8.80 ± 0.82	9.06 ± 0.38
	F	8.78 ± 1.12	9.60 ± 1.13	9.00 ± 0.69	9.17 ± 0.75
**MCV** (fL)	M	56.10 ± 4.59	54.33 ± 5.01	52.83 ± 3.87	52.600 ± 2.60
	F	56.39 ± 3.75	49.50 ± 6.36	54.17 ± 3.31	53.17 ± 4.70
**MCHC** (g/dL)	M	29.50 ± 2.76	30.22 ± 1.78	31.60 ± 0.97	31.0 ± 1.25
	F	29.52 ± 2.65	32.20 ± 1.69	31.33 ± 1.15	30.50 ± 1.52
**PLT** (K/μL)	M	1580 ± 736	746.40 ± 262.67	856.80 ± 67.43	981 ± 175.89
	F	1494 ± 769	840*	540.00 ± 338.33	792 ± 334.13
**WBC** (K/μL)	M	8.79 ± 2.54	5.17 ± 1.86	6.85 ± 3.42	6.64 ± 0.95
	F	8.77 ± 2.95	8.70 ± 2.26	6.18 ± 1.80	7.63 ± 1.71
**Neutrophils** (K/μL)	M	1.50 ± 0.84	1.23 ± 0.14	2.52 ± 1.33	1.90 ± 1.11
	F	1.34 ± 0.73	1.75 ± 0.64	1.23 ± 0.43	1.26 ± 0.74
**Lymphocites** (K/μL)	M	6.59 ± 1.90	3.68 ± 1.77	3.98 +/- 2.04	4.44 +/- 1.20
	F	6.70 ± 2.17	6.60 ± 1.41	4.63 ± 1.37	6.00 ± 1.11
**Monocytes** (K/μL)	M	0.49 ± 0.19	0.22 ± 0.15	0.27 ± 0.15	0.24 ± 0.10
	F	0.50 ± 0.21	0.25 ± 0.07	0.30 ± 0.26	0.25 ± 0.15

Each value represents the Average ± SD; n=5 #https://www.criver.com/products-services/find-model/cd-1r-igs-mouse?region=3616. HCT: Hematocrit. HGB: Hemoglobin. RBC: Red blood cell. MCV: Mean Corpuscular Volume. MCHC: Mean corpuscular hemoglobin concentration. PLT: Platelet. WBC: White Blood Cells. M: males. F: Females. * Only one value was obtained in parament. Single value.

Blood chemistry:

The values of 16 analytes in blood samples were measured. Statistically significant differences were observed in total protein, globulins, calcium and phosphorus in females of all groups, and calcium and phosphorus in males. No significant differences were observed in the quantification of the other parameters evaluated. The results of the blood chemistry evaluation are shown in the following table.

Table S5. Blood chemistry summary in CD-1 mice, 14 days i.v. post-administration of IgG-A7.

Blood parameter	Sex	Ref. values*	Control	100 mg/Kg	200 mg/Kg
**Glucose** (mg/dL)	M	185-270	243.90 ± 39.60	249.50 ± 27.00	232.20 ± 23.40
	F	207-320	245.00 ± 22.32	222.66 ± 1.75	216.90 ± 28.44
**Urea** (mg/dL)	M	23-43	24 ± 4.0	28 ± 3.0	27 ± 1.0
	F	21-35	26 ± 5.0	24 ± 3.0	28 ± 6.0
**Creatinine** (mg/dL)	M	0.2-0.8	0.55 ± 0.23	0.51 ± 0.17	0.57 ± 0.09
	F	0.4-0.8	0.56 ± 0.22	0.46 +/-0.11	0.61 +/- 0.12
**Cholesterol** (mg/dL)	M	31-91	61.02 ± 14.76	66.60 ± 11.34	64.62 ± 12.06
	F	41-77	57.42 ± 10.62	44.82 ± 7.02	55.44 ± 6.48
**Triglycerides** (mg/dL)	M	37.3 ± 0.4	35.46 ± 4.68	34.20 ± 8.64	44.64 ± 21.96
	F	71.3 ± 6.3	35.46 ± 6.12	37.98 ± 6.30	41.94 ± 21.24
**Total bilirubins** (mg/dL)	M	0.0-0.3	0.02 ± 0.02	0.03 ± 0.02	0.02 ± 0.01
	F	0-0.5	0.01 ± 0.004	0.03 ± 0.01	0.03 ± 0.01
**ALT** (IU/L)	M	28-190	57.50 ± 11.50	92.83 ± 14.20	102.00 ± 71.50
	F	56-159	83.00 ± 24.40	83.83 ± 22.40	98.50 ± 32.60
**AST** (IU/L)	M	24-472	190.20 ± 102.60	243.20 ± 40.10	180.00 ± 119.02
	F	87-187	358.0 ± 183.90	228.83 ± 60.00	261.42 ± 117.00
**AP** (IU/L)	M	86-246	99.00 ± 25.20	81.70 ± 20.22	113.00 ± 20.99
	F	118-206	117.50 ± 48.17	103.80 ± 16.77	117.60 ± 37.40
**CK** (IU/L)	M	0-1800[#]	1800 ± 1522.10	2035 ± 802.84	1481 ± 2211.40
	F	0-2021[#]	3270 ± 1758.60	2005 ± 983.04	1693 ± 2388.50
**Total proteins** (g/dL)	M	3.0-5.6	5.58 ± 1.02	5.67 ± 0.97	5.38 ± 0.56
	F	4.2-5.2	6.90 ± 1.34[≠]	5.57 ± 0.63[≠]	5.87 ± 0.33[≠]
**Albumin** (g/dL)	M	1.8-3.4	2.92 ± 0.07	2.98 ± 0.07	2.83 ± 0.23
	F	2.9-3.7	3.50 ± 0.77	3.35 ± 0.22	3.23 ± 0.29
**Globulins** (g/dL)	M	1.2-2.2	2.27 ± 0.97	2.98 ± 0.07	2.55 ± 0.52
	F	1.2-1.6	3.40 ± 0.88[≠]	3.35 ± 0.23[≠]	2.63 ± 0.43[≠]
**A/G ratio**	M	1.3-1.9	1.18 ± 0.29	1.21 ± 0.32	1.15 ± 0.26
	F	1.03-1.57	1.07 ± 0.28	1.54 ± 0.20	1.26 ± 0.29
**Calcium** (mg/dL)	M	12.0-12.4	39.24 ± 7.38[≠]	31.14 ± 12.24[≠]	26.46 ± 12.41[≠]
	F	8.4-10.2	19.44 ± 2.16[≠]	32.04 ± 7.74[≠]	23.22 ± 10.08[≠]
**Phosphorus** (mg/dL)	M	7.8-18.6	50.04 ± 7.38[≠]	47.34 ± 7.02[≠]	51.30 ± 3.78[≠]
	F	7.5-15.0	57.78 ± 6.66[≠]	51.66 ± 5.22[≠]	51.48 ± 7.92[≠]

Each value represents the mean +/- SD; n≥ 4. * The Clinical Chemistry of Laboratory Animals. Richard H. Luong. Third Edition. [#] C57 mice values reference. ALT: Alanine Transaminase; AST: Aspartate Aminotransferase; AP: Alkaline phosphatase; CK: creatine kinase. [≠] Values outside reference range. M, males; F, Females.

Mouse Tissue Cross-reactivity:

IgG-A7 cross-reactivity using 13 mouse tissues. No cross-reactivity with any of the tissues evaluated was observed.

Table S6. Evaluation of mice tissues exposed to IgG1-A7-FITC (0.625 μg/mL).

Tissue Type	Replicates	Age (weeks)	Gender	Diagnosis	Specific Positive Staining (D1T-FITC):		Location	Tissue Integrity Acceptable
					I	D		
**HEK293T (SARS-CoV-2 Spike)**	1	NA	NA	NA	3	++	Cytoplasmic and membranous	
**HEK293T (No SARS-CoV-2 Spike)**	1	NA	NA	NA	0	-	-	
**Adrenal 1**	3	6-8	M	N	0	-	- LS	Y
		6-8	F	N	0	-	-	Y
		6-8	M	N	0	-	-	Y
**Brain, Cerebellum**	3	6-8	M	N	0	-	-	Y
		6-8	M	N	0	-	-	Y
		6-8	F	N	0	-	-	Y
**Brain, Cerebrum**	3	6-8	F	N	0	-	-	Y
		6-8	F	N	0	-	-	Y
		6-8	M	N	0	-	-	Y
**Breast**	3	6-8	F	N	0	-	-	Y
		6-8	M	N	0	-	- LS	Y
		6-8	F	N	0	-	- LS	Y
**Colon**	3	6-8	F	N	0	-	-	Y
		6-8	M	N	0	-	-	Y
		6-8	M	N	0	-	-	Y
**Esophagus**	3	6-8	F	N	0	-	-	Y
		6-8	M	N	0	-	-	Y
		6-8	F	N	0	-	-	Y
**Heart 1**	3	6-8	M	N	0	-	-	Y
		6-8	F	N	0	-	-	Y
		6-8	F	N	0	-	-	Y
**Kidney 1**	3	6-8	F	N	0	-	-	Y
		6-8	F	N	0	-	-	Y
		6-8	M	N	0	-	-	Y
**Liver 1**	3	6-8	F	N	0	-	-	Y
		6-8	F	N	0	-	-	Y
		6-8	M	N	0	-	-	Y
**Lung 1**	3	6-8	F	N	0	-	-	Y
		6-8	M	N	0	-	-	Y
		6-8	F	N	0	-	-	Y
**Skeletal Muscle**	3	6-8	F	N	0	-	-	Y
		6-8	F	N	0	-	-	Y

		6-8	M	N	0	-	-	Y
**Sciatic Nerve**	3	6-8	F	N	0	-	-	Y
		6-8	M	N	0	-	-	Y
		6-8	F	N	0	-	-	Y
**Ovary**	3	6-8	F	N	0	-	-	Y
		6-8	F	N	0	-	-	Y
		6-8	F	N	0	-	-	Y

M: males. F: Females. N: Normal. LS: Limited Sample. Y: Yes. I: Intensity. D: Distribution

Human Tissue Cross-reactivity:

IgG-A7 cross-reactivity using 34 relevant human tissues. No cross-reactivity with any of the tissues evaluated was observed.

Table S7. Evaluation of human tissues. The IgG1-A7 antibody was tested at optimal (0.625 μg/mL) supra-optimal (1.25 μg/mL) nor sub-optimal (0.078 μg/mL) concentrations.

Tissue	Result							Specific Positive staining	Tissue Integrity acceptable
	Replicate	I	D	I	D	I	D		
**HEK293T (SARS-CoV-2 Spike)**	1	3	++	3	++	2	++	Withing cells (Cytoplasmic and membranous)	NA
	2	3	++	3	++	1	+		NA
**HEK293T (SARS-CoV-2 Spike)**	1	0	-	0	-	0	-	Not seen	NA
	2	0	-	0	-	0	-		NA
**Adrenal Gland**	1	0	-	0	-	0	-	Not seen (LS)	Y
	2	0	-	0	-	0	-	Not seen	Y
	3	0	-	0	-	0	-	Not seen	Y
**Blood Cells**	1	0	-	0	-	0	-	Not seen	NA
	2	0	-	0	-	0	-	Not seen	NA
	3	0	-	0	-	0	-	Not seen	NA
**Bone Marrow**	1	0	-	0	-	0	-	Not seen	Y
	2	0	-	0	-	0	-	Not seen	Y
	3	0	-	0	-	0	-	Not seen (LS)	Y
**Brain (Cerebellum)**	1	0	-	0	-	0	-	Not seen	Y
	2	0	-	0	-	0	-	Not seen	Y
	3	0	-	0	-	0	-	Not seen	Y
**Breast**	1	0	-	0	-	0	-	Not seen	Y
	2	0	-	0	-	0	-	Not seen	Y
	3	0	-	0	-	0	-	Not seen	Y
**Eye**	1	0	-	0	-	0	-	Not seen	Y
	2	0	-	0	-	0	-	Not seen	Y
	3	0	-	0	-	0	-	Not seen	Y
**Fallopian Tube**	1	0	-	0	-	0	-	Not seen	Y
	2	0	-	0	-	0	-	Not seen	Y
	3	0	-	0	-	0	-	Not seen	Y
**Stomach**	1	0	-	0	-	0	-	Not seen	Y
	2	0	-	0	-	0	-	Not seen	Y
	3	0	-	0	-	0	-	Not seen	Y
**Small Intestine**	1	0	-	0	-	0	-	Not seen	Y
	2	0	-	0	-	0	-	Not seen	Y
	3	0	-	0	-	0	-	Not seen	Y
**Large Intestine**	1	0	-	0	-	0	-	Not seen	Y
	2	0	-	0	-	0	-	Not seen	Y
	3	0	-	0	-	0	-	Not seen	Y
**Heart**	1	0	-	0	-	0	-	Not seen	Y
	2	0	-	0	-	0	-	Not seen	Y
	3	0	-	0	-	0	-	Not seen	Y
**Kidney**	1	0	-	0	-	0	-	Not seen	Y

	2	0	-	0	-	0	-	Not seen	Y
	3	0	-	0	-	0	-	Not seen	Y
**Liver**	1	0	-	0	-	0	-	Not seen	Y
	2	0	-	0	-	0	-	Not seen	Y
	3	0	-	0	-	0	-	Not seen	Y
**Lung**	1	0	-	0	-	0	-	Not seen	Y
	2	0	-	0	-	0	-	Not seen	Y
	3	0	-	0	-	0	-	Not seen (LS)	Y
**Lymph Node**	1	0	-	0	-	0	-	Not seen	Y
	2	0	-	0	-	0	-	Not seen	Y
	3	0	-	0	-	0	-	Not seen	Y
**Ovary**	1	0	-	0	-	0	-	Not seen	Y
	2	0	-	0	-	0	-	Not seen	Y
	3	0	-	0	-	0	-	Not seen	Y
**Pancreas**	1	0	-	0	-	0	-	Not seen	Y
	2	0	-	0	-	0	-	Not seen	Y
	3	0	-	0	-	0	-	Not seen	Y
**Peripheral Nerve**	1	0	-	0	-	0	-	Not seen	Y
	2	0	-	0	-	0	-	Not seen	Y
	3	0	-	0	-	0	-	Not seen	Y
**Parotid Salivary Gland**	1	0	-	0	-	0	-	Not seen	Y
	2	0	-	0	-	0	-	Not seen	Y
	3	0	-	0	-	0	-	Not seen	Y
**Pituitary Gland**	1	0	-	0	-	0	-	Not seen	Y
	2	0	-	0	-	0	-	Not seen (LS)	Y
	3	0	-	0	-	0	-	Not seen	Y
**Placenta**	1	0	-	0	-	0	-	Not seen	Y
	2	0	-	0	-	0	-	Not seen	Y
	3	0	-	0	-	0	-	Not seen	Y
**Prostate Gland**	1	0	-	0	-	0	-	Not seen	Y
	2	0	-	0	-	0	-	Not seen	Y
	3	0	-	0	-	0	-	Not seen	Y
**Skin**	1	0	-	0	-	0	-	Not seen	Y
	2	0	-	0	-	0	-	Not seen	Y
	3	0	-	0	-	0	-	Not seen	Y
**Spinal Cord**	1	0	-	0	-	0	-	Not seen	Y
	2	0	-	0	-	0	-	Not seen	Y
	3	0	-	0	-	0	-	Not seen	Y
**Spleen**	1	0	-	0	-	0	-	Not seen	Y
	2	0	-	0	-	0	-	Not seen	Y
	3	0	-	0	-	0	-	Not seen	Y
**Striated Muscle**	1	0	-	0	-	0	-	Not seen	Y
	2	0	-	0	-	0	-	Not seen	Y
	3	0	-	0	-	0	-	Not seen	Y
**Testis**	1	0	-	0	-	0	-	Not seen	Y
	2	0	-	0	-	0	-	Not seen	Y
	3	0	-	0	-	0	-	Not seen	Y
**Thymus**	1	0	-	0	-	0	-	Not seen	Y
	2	0	-	0	-	0	-	Not seen	Y
	3	0	-	0	-	0	-	Not seen	Y
**Thyroid Gland**	1	0	-	0	-	0	-	Not seen	Y

	2	0	-	0	-	0	-	Not seen	Y
	3	0	-	0	-	0	-	Not seen (LS)	Y
**Tonsil**	1	0	-	0	-	0	-	Not seen	Y
	2	0	-	0	-	0	-	Not seen	Y
	3	0	-	0	-	0	-	Not seen	Y
**Ureter**	1	0	-	0	-	0	-	Not seen	Y
	2	0	-	0	-	0	-	Not seen	Y
	3	0	-	0	-	0	-	Not seen	Y
**Urinary Bladder**	1	0	-	0	-	0	-	Not seen	Y
	2	0	-	0	-	0	-	Not seen	Y
	3	0	-	0	-	0	-	Not seen	Y
**Uterus (Cervix)**	1	0	-	0	-	0	-	Not seen	Y
	2	0	-	0	-	0	-	Not seen	Y
	3	0	-	0	-	0	-	Not seen	Y
**Uterus (Endometrium)**	1	0	-	0	-	0	-	Not seen	Y
	2	0	-	0	-	0	-	Not seen	Y
	3	0	-	0	-	0	-	Not seen	Y

LS: Limited Sample. NA: Not applicable. Y: Yes I. I: Intensity. D: Distribution.
